# Determination of Dimple Core Design Configurations for Sandwich Panel Enhancement Using Fuzzy-Hybrid Decision Making Analysis

**DOI:** 10.3390/ma16030935

**Published:** 2023-01-18

**Authors:** Mohd Khairul Faidzi, Shahrum Abdullah, Salvinder Singh Karam Singh, Mohamad Faizal Abdullah, Abdul Hadi Azman

**Affiliations:** 1 Department of Mechanical and Manufacturing Engineering, Faculty of Engineering and Built Environment, Universiti Kebangsaan Malaysia, UKM Bangi 43600, Selangor, Malaysia; 2 Department of Mechanical Engineering, Faculty of Engineering, Universiti Pertahanan Nasional Malaysia, Kem Perdana Sg. Besi, Sungai Besi 57000, W.P. Kuala Lumpur, Malaysia

**Keywords:** metal sandwich panel, finite element analysis, core design, critical criteria, structural integrity

## Abstract

The purpose of this paper is to determine the best dimple core design for metal sandwich panels by investigating the various critical criteria and core design parameters using the fuzzy-hybrid multi-criteria decision-making tool. The structural integrity of a sandwich panel depends on the core design and significantly affects the bonding strength. The continuous design and testing of a sandwich panel is a very lengthy process that increases the design time. The simulation analysis output was segregated into nine critical failure criteria. All the critical criteria weightages were evaluated using the Fuzzy-Analytical Hierarchical Process, while the Fuzzy—Technique for Order Preference by Similarity to Ideal Solution was used to evaluate the Closeness Coefficient value to determine the best core design configuration. The results indicate that the core configuration with a diameter of 6.0 mm and a depth of 3.0 mm obtained the highest closeness coefficient values, 0.9937 and 0.9294, under cyclic loading conditions of 50% and 70%. It was shown that using average sizes in the dimple configuration tends to provide better delamination resistance and structural integrity. This study contributes to the selection of the optimum core design configuration based on the various design criteria and using non-complex and competent analysis.

## 1. Introduction

The application of sandwich panels in many branches of the engineering industry, such as vehicle manufacturing, has expanded due to their special characteristics such as lightness, good energy absorption and impact resistance [1]. However, the configuration of a sandwich panel, especially in terms of the core design, is crucial. The design must be based on the types of application and the loading given, such as continuous cyclic loading or impact loading [2,3]. Various methods can be used in the joining mechanisms for sandwich panels, such as welding, brazing, adhesive joints and stitching [4]. Recent core designs used for sandwich panels include solid plate, foam, honeycomb and lattice. Previous literature [5,6] has identified how most recent cores—such as foam, honeycomb and lattice—have cavities and tend to suffer catastrophic failure when subjected to extreme loading conditions such as cyclic loading. The core design of a panel has a significant impact in terms of the distribution of bonding strength and the stress on the bonding layer. Additional modifications to the core surface, material properties and panel configurations could enhance the bonding resistance and produce better holding strength between the panel layers [7]. It has been reported that changing the multilayer factor for laminated panels enhances the core strength and structural integrity of the panels [8]. This also involves various criteria that need to be considered, such as the stress distribution, energy absorption, deformation and fatigue performance, along with the design parameters such as the diameter, depth, core thickness and angle of the force being applied [9,10]. Hence, further analysis must be performed via a mathematical approach to resolve this engineering design-based problem without the need for a time-consuming process at the design stage [11].

M-criteria decision-making (MCDM) analysis has become an important tool to determine ideal solutions, especially when various elements regarding criteria and alternatives are involved [12]. The element of triangular fuzzy numbers (TFNs) has been used to enhance the precision and accuracy of design selection-based problems [13,14]. The most widely established techniques in the MCDM method are the Fuzzy-Analytical Hierarchical Process (F-AHP) and the Fuzzy-Technique for Order Preference by Similarity to Ideal Solution (F-TOPSIS), as they are capable of considering various criteria and design alternatives to produce a highly accurate and robust design solution [15]. Using the combination of the output from finite element analysis (FEA) and the fuzzy-hybrid MCDM method, all the critical criteria of the alternative designs were evaluated without bias perception in F-AHP and equally through the Euclidean-based distance analysis in F-TOPSIS. Based on the previous literature, the Euclidean-based distance analysis in F-TOPSIS is displayed in the ranking system, and any value close to 1.0 represents the ideal solution to the design-based problem [16]. In other words, the optimum engineering design can be determined mathematically without involving extra production costs. However, the relationship between the core design parameter with the factors that lead to failure determination is vague and additional work is needed to correlate this intertwined connection. Hence, the connection between the FEA results and the optimised analysis regarding the core design remains vague and needs to be explored further.

Sandwich panel failure analysis usually involves the analysis of material properties and behaviours, such as ductility and flexural behaviours, especially at the joining area [17]. Previous studies revealed that FEA has been used to analyse sandwich panels, especially the influence of the size cell and the configuration of the core panel inside the sandwich panel [18,19]. The literature suggests that most researchers have used F-AHP and F-TOPSIS to indicate the optimum selection of the design parameters for a certain application, such as the material selection and machining conditions [20]. In F-AHP and F-TOPSIS, there is a mechanism to verify the stability of the developed comparison matrix and normalise the matrix tables, but it is still compulsory to verify the optimised results from a different source, such as the failure plot diagram of a sandwich panel. In that way, the intertwined relationships between FEA and the various design elements could be used to determine the most robust core design [10,21]. Hence, it is vital to introduce an alternative method that includes a combination of the two established methods (FEA and hybrid MCDM) to maximise the benefits of the analysis results and verify the optimum analysis in a simplified way. This important step must be taken since it contributes to the structural integrity analysis of a sandwich panel, especially in terms of verifying the optimum core design configuration.

The core design configuration plays a significant role in enhancing the mechanical performance of a sandwich panel in terms of delamination resistance. The recent core design configuration has cavities in its structure, leading to unstable bonding structural integrity between the panel layers. Hence, it is vital to develop an optimum core design configuration to prevent early debonding or delamination phenomena. Therefore, this study aimed to optimise the core design using simulation analysis of a four-point bending setup, based on the ASTM C393 at the maximum cyclic loading conditions of 50% and 70%. The simulation analysis output was determined and further analysed using fuzzy-hybrid MCDM, F-AHP and F-TOPSIS. The combination of FEA and fuzzy-hybrid MCDM analysis was important in determining the optimum core design results. The optimised results were then verified using a failure plot diagram of the hotspot region of the core panel. Hypothetically, the large area of hemispherical dimple core design would reduce the bonding strength at the joining region and increase the chances of any early delamination phenomena. This study makes three main contributions that are worth exploring further. Firstly, it contributes to the investigation of the intertwined relationship between the various elements of critical parameters and the different core design alternatives, with the hybrid MCDM method and the element of triangular fuzzy numbers (TFNs) used to produce an accurate and effective design selection solution. Secondly, the study reveals the possibility of an integrated method featuring the finite element analysis (FEA) output and the hybrid MCDM method to rapidly solve problems in the manufacturing design process. Finally, the study provides a novel enhancement to the core panel configuration in terms of improving the delamination resistance via modifications to the core surface configuration.

## 2. Materials and Methods

**Figure 1:**
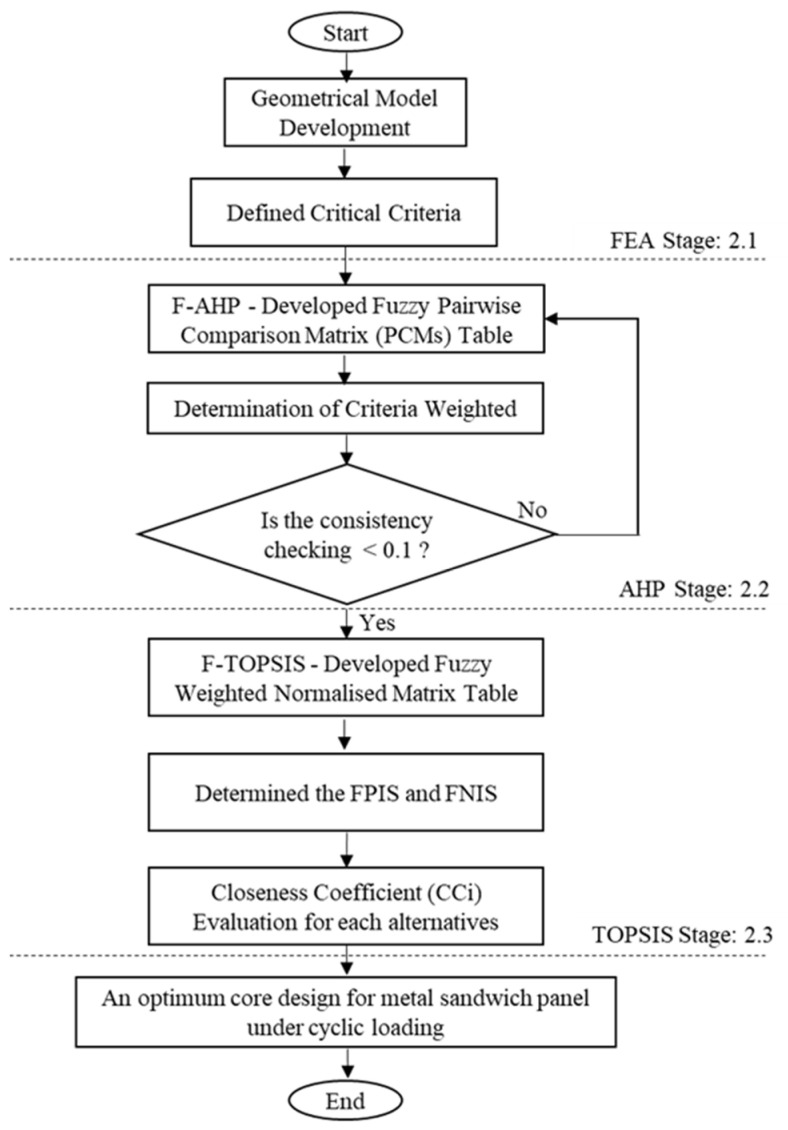
The overall study process flow.

Three-dimensional geometrical sandwich panels were developed using a finite element software package. A four-point bending setup, according to the ASTM C393 [22], was used in the simulation to gather data such as the stress distribution, deformation, fatigue life and damage values. The core panels of the sandwich panel sample were modified into four different diameters and depth dimensions in dimple core design configuration. Figure 1 shows the overall study process flow. Based on the FEA input, the main criteria contributing to the performance of the sandwich panels were evaluated and further analysed via hybrid F-MCDM with TFNs. The aim was to evaluate and justify the best dimple configuration core design under fatigue bending conditions using a four-point bending setup.

### 2.1. Categorisation of Critical Criteria from Finite Element Analysis

**Figure 2:**
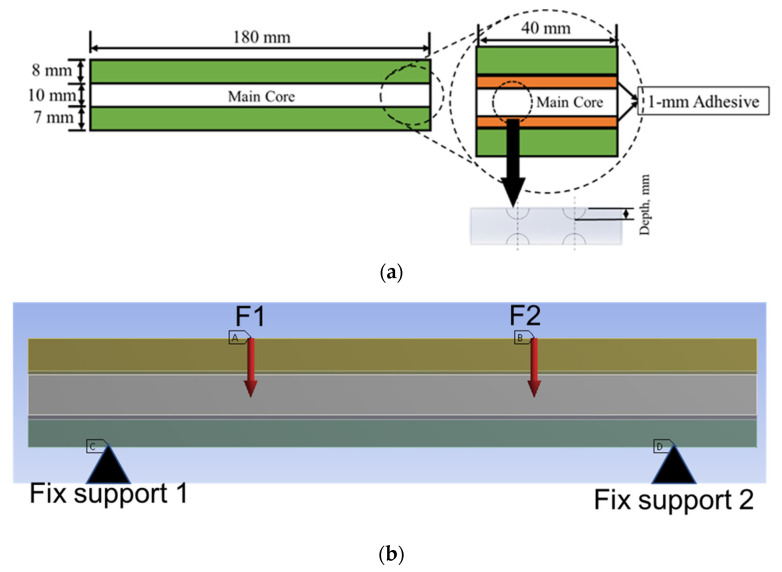
The sketch and setup of a geometrical panel (**a**) with its dimensions and (**b**) in the four-point bending simulation.

The sandwich panels were subjected to 50% and 70% of cyclic loading conditions to observe their performance characteristics such as the stress distribution, deformation, fatigue life and damage distribution on the core panel region. Through the simulated results, the main failure criteria contributing to the sandwich panel performance were determined and defined, as shown in Table 1. A geometrical panel was developed with a length of 180 mm, a width of 40 mm and an overall thickness of 27 mm, including their 1-mm thickness at the upper and lower layers of the bonding region, as shown in Figure 2. In terms of the hemispherical core design, the four dimple core design configuration alternatives (different diameters and depths) were analysed in this study. Epoxy resin with hardener was employed as the bonding material for the metal sandwich panels.

**Figure 3:**
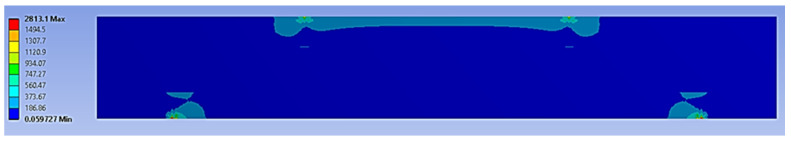
Example of stress distribution under the four-point bending simulation.

**Figure 4:**
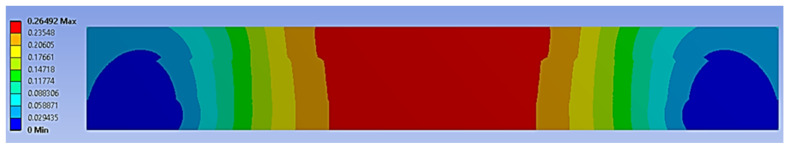
Example of total deformation distribution under the four-point bending simulation.

The four-point bending simulation was conducted based on the ASTM C393, which is suitable for characterising the behaviour of sandwich panels using a variety of core configurations [10,22]. The loading (denoted as F1 and F2) given to the sandwich panels was determined using Equation (1) [10]. However, to preserve the structural integrity of each core and sandwich panel, the maximum stress used in Equation (1) was selected from the weakest material properties of the sandwich panels. In this case, it was the magnesium alloy AZ31B, since this is the lightest material used as a sandwich panel core. The simulation was subjected to static loading under 70% and 50% of maximum force loading. For the simulation under fatigue loading conditions, the Goodman fatigue stress life theory was selected to indicate the optimum fatigue life performance of the panel. In addition, variable amplitude loading (VAL) data were obtained using the customised analytical process. The equivalent of this was identified in terms of the damage values, with specific software used to produce the equivalent constant amplitude loading (CAL) data. The equivalent damage process was important to preserve the accuracy and stability of the simulation results [23]. The CAL and VAL data were used in the simulation to observe the effects of each dimple core configuration variation in terms of fatigue life and damage distribution. Through the responses from the simulation under four-point bending conditions, as shown in Figure 3 and Figure 4, nine significant criteria were determined and selected for further study using F-MCDM

(1) $$\sigma_{max,\;x}=\frac{1}{12}FL\;.\;\frac{1}{2}h\;.\;\left(-\frac{12}{bh^{3}}\right)=\frac{FL}{-2bh^{2}}$$

where $\sigma_{max,\;x}$ is the maximum stress from the weakest material in the sandwich panel, *F* is the force given to the panel, *L* is the total length of the panel, *b* is the total width of the panel and *h* is the total height of the panel.

**Figure 5:**
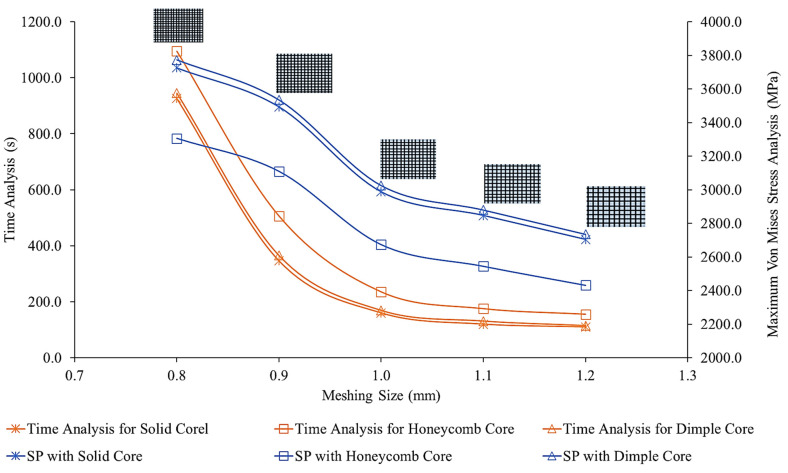
The mesh sensitivity analysis for meshing sizes between 0.8 to 1.2 mm, with different core designs shown for comparison.

Mesh sensitivity analysis was conducted to ensure the stability and accuracy of the simulation. The mesh sensitivity analysis outcomes were converged using a 64-bit operating system with Intel Core i7 and 32 GB random access memory (RAM). Figure 5 shows the mesh sensitivity analysis, based on the analysis of one failure criterion (von Mises stress), along with the time taken for the analysis. Based on this figure, it was found that a mesh size of 1.0 mm was sufficient to be used in the simulation due to the average stability and simulation time. In addition, a mesh size between 1.0 and 3.0 mm was expected to be sufficient to provide an accurate simulation analysis in a reasonable simulation time [3,10]. Lowering the mesh size required more RAM space and high-tech computer accessories [2,10]. Table 2 shows the total number of nodes and elements for the three-dimensional sandwich panel models with different dimple core configurations.

### 2.2. Defining the Nine Main Criteria Weight Using the Fuzzy Analytical Hierarchical Process (F-AHP)

**Figure 6:**
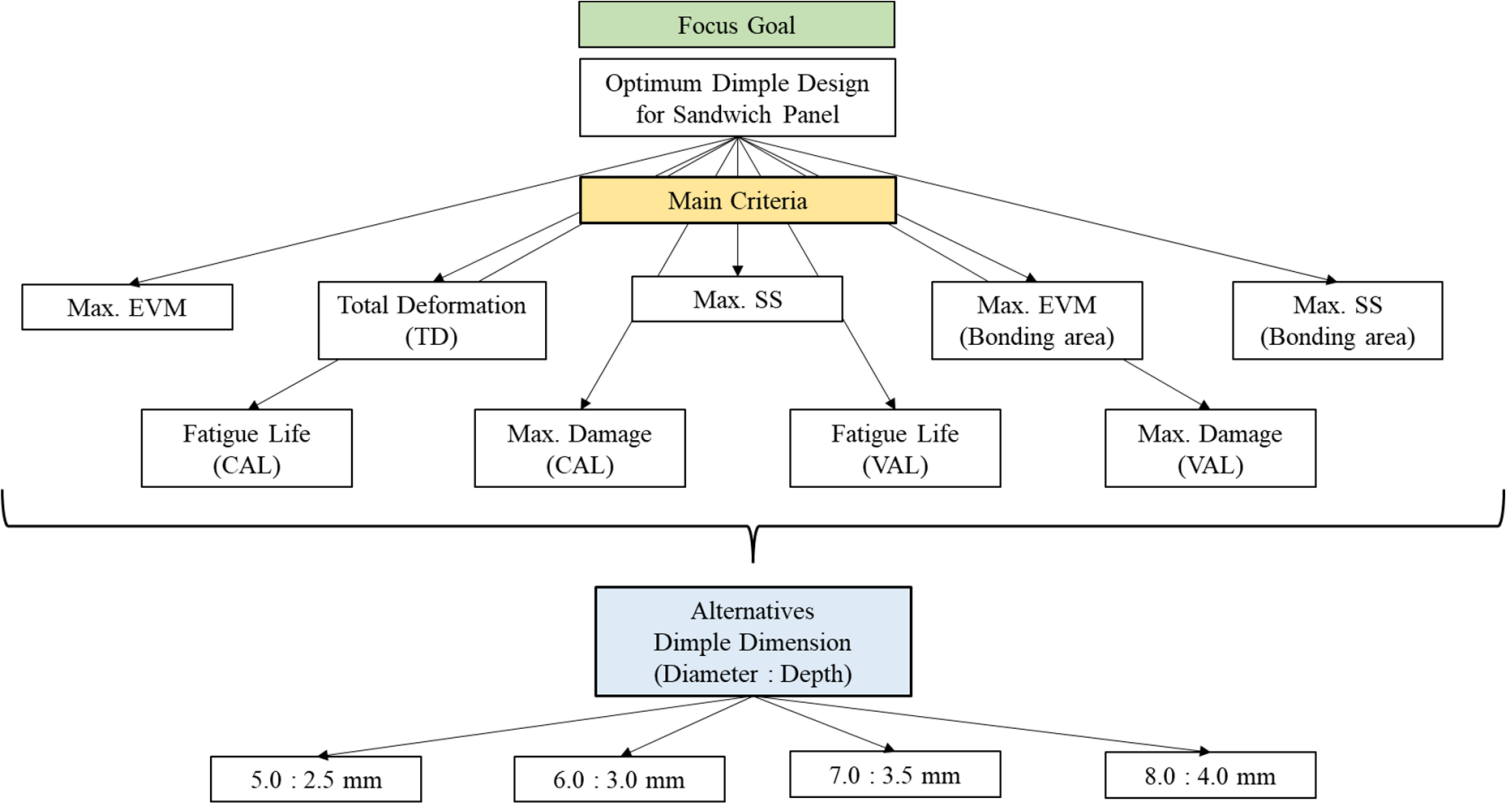
The developed hierarchical tree determined from finite element analysis (FEA).

The nine significant criteria determined after the simulation process were studied further using F-MCDM. In the second stage, the fuzzified weights for each criterion were evaluated using the F-AHP. The TFNs were used in this F-AHP method as this is the most established and least complicated method of determining the weightages of the criteria in the F-AHP [24,25]. Figure 6 shows the hierarchical tree that was constructed with the nine important criteria and four different core design alternatives, based on the FEA results.

The crisp values in the pairwise comparison matrix (PCMs) table were determined based on the researcher’s previous evaluation of the FEA results. All the crisp values in the PCMs table were sensitivity-checked, using Equation (2) for the consistency index (*CI*) and Equation (3) for the consistency ratio (*CR*).

Sensitivity checking is vital at the F-AHP stage to ensure that all the crisp value data in the PCMs table has no bias perception and to increase the trustworthiness of the values assigned in the PCMs table [26].

(2) $$CI=\frac{\lambda_{max}-n}{n-1},$$

(3) $$CR=\frac{CI}{RI},$$

The random index (*RI*) value used in Equation (3) was determined as illustrated in Table 3. For the criteria weight analysis, the PCMs table was first developed using the crisp values of the Saaty’s Scale, as shown in Table 4.

Once the consistency had been checked and it had been shown that the crisp AHP value data in the PCMs table accorded to values below 0.1, the triangular fuzzified PCMs table was constructed. In other words, all the crisp AHP values in the PCMs table were converted into TFNs, as shown in Table 4, to form a new triangular fuzzified pairwise comparison matrix (F-PCMs) table to evaluate the fuzzified weightages for all the criteria.

The Saaty’s Scale with the F-AHP scale, as shown in Table 4, was important to determine the robustness crisp values in the PCMs table before the fuzzy element was infused on each value in that table [14,15]. The reciprocal triangular fuzzy scale in the F-PCMs table was evaluated using Equation (4) [25]:

(4) $$Reciprocal\;TFS=\left(\frac{1}{\sum_{i=1}^{n}u_{i}},\frac{1}{\sum_{i=1}^{n}m_{i}},\frac{1}{\sum_{i=1}^{n}l_{i}}\right),$$

where *u_i_* refers to the upper value, *m_i_* refers to the middle value and *l_i_* refers to the lower value in the triangular fuzzy numbers element.

As Table 4 shows, all the values in the PCMs table were evaluated to evaluate the weights of the decision criteria in triangular fuzzified form, as shown in Equation (5) [25]:

(5) $$\tilde{A}=\begin{array}{c} C_{1}\\ C_{2}\\ \vdots \\ C_{n} \end{array}\left[\begin{array}{cccc} C_{1} & C_{2} & \ldots & C_{n}\\ 1 & \left(l_{12},m_{12},u_{12}\right) & \ldots & \left(l_{1n},m_{1n},u_{1n}\right)\\ \left(\frac{1}{u_{21}},\frac{1}{m_{21}},\frac{1}{l_{21}}\right) & 1 & \ldots & \left(l_{2n},m_{2n},u_{2n}\right)\\ \vdots & \vdots & \vdots & \vdots \\ \left(\frac{1}{u_{1n}},\frac{1}{m_{1n}},\frac{1}{l_{1n}}\right) & \left(\frac{1}{u_{2n}},\frac{1}{m_{2n}},\frac{1}{l_{2n}}\right) & \ldots & 1 \end{array}\right],$$

where the main criteria are denoted as *C*_1_, …, *C_n_* and $\tilde{A}$ is denoted as the F-PCMs table.

Once the F-PCMs criteria table had been established using Equation (4), the fuzzified weightage of each criterion was identified using the fuzzy geometric mean values obtained via Equations (6)–(8) [16]:

(6) $$\tilde{r_{i}}={\left[{\tilde{a}}_{i1}⊗\ldots ⊗{\tilde{a}}_{in}\right]}^{1/n},$$

where $\tilde{r_{i}}$ is the fuzzy geometric mean for comparison purposes.

(7) $${\left(\overset{n}{\underset{i}{\sum }}{\tilde{r}}_{i}\right)}^{-1}={\left({\tilde{r}}_{i1}⊕{\tilde{r}}_{i2}⊕\ldots ⊕{\tilde{r}}_{in}\right)}^{-1},$$

where ${\left(\sum_{i}^{n}{\tilde{r}}_{i}\right)}^{-1}$ is the inverse (−1) power of the summation vector of the fuzzy geometric mean.

(8) $${\tilde{w}}_{i}={\tilde{r}}_{i\;}⊗{\left({\tilde{r}}_{i1}⊕{\tilde{r}}_{i2}⊕\ldots ⊕{\tilde{r}}_{in}\right)}^{-1}$$

where ${\tilde{w}}_{i}$ is the relative fuzzy weight for each criterion denoted by *i*.

**Figure 7:**
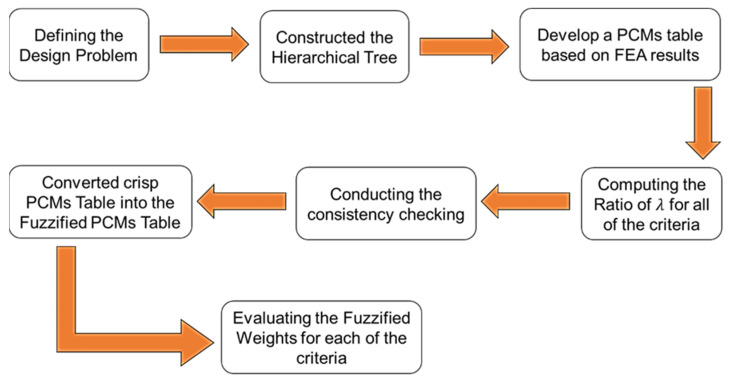
The main steps to determine the fuzzified weights for each criterion using the F-AHP method.

The process flow to determine the weights for each criterion using the F-AHP method is illustrated in Figure 7. Through the process flow, it was possible to develop a robust and consistent F-PCMs table by performing consistency checking on the crisp value PCMs before the conversion into the F-PCMs table [14,28]. The fuzzified weights determined using the F-AHP were then used for the next analysis to justify the best dimple core design configuration.

### 2.3. Determining the Best Dimple Core Design Configuration Using Fuzzy Technique for Order Preference by Similarity to Ideal Solution (F-TOPSIS)

In the third stage, all the fuzzified weights for each criterion that had been determined through the F-AHP method were infused into F-TOPSIS. The fuzzified weighted normalised decision matrix (F-DMs) table was developed using Equation (9) [29]:

(9) $${\tilde{v}}_{ij}={\tilde{r}}_{ij}\cdot {\tilde{w}}_{ij}$$

where ${\tilde{v}}_{ij}\;$ denotes the fuzzified weighted normalised value, ${\tilde{r}}_{ij}\;$ was assigned as the normalised criteria value whilst ${\tilde{w}}_{ij}\;$ represents the weight for each criterion, as determined through the F-AHP method.

Through the F-DMs table, the fuzzy positive and negative ideal solutions, known as FPIS and FNIS, were determined. Usually, the FPIS values are the preferred values of the criteria in the F-DMs table, whilst the FNIS values are the least preferable values of the criteria in the F-DMs table [29,30]. However, some criteria in this study—such as the deformation and damage values—tended to have a minimal value for the ideal solution and were preferred for selection as the FPIS value rather than the FNIS value, since the minimal value is the most favourable for these criteria.

The FPIS and FNIS values were determined using Equations (10) and (11) [29]:

(10) $$A^{+}=\left\{{\tilde{v}}_{1}^{+},{\tilde{v}}_{2}^{+},\ldots ,{\tilde{v}}_{n}^{+}\right\}=\left\{\left(max_{j}v_{ij}|i\in B\right),\left(min_{j}v_{ij}|i\in C\right)\;\right\},$$

(11) $$A^{-}=\left\{{\tilde{v}}_{1}^{-},{\tilde{v}}_{2}^{-},\ldots ,{\tilde{v}}_{n}^{-}\right\}=\left\{\left(min_{j}v_{ij}|i\in B\right),\left(max_{j}v_{ij}|i\in C\right)\;\right\},$$

where ${\tilde{v}}_{n}^{+}$ represents the maximum value of *i* for all alternatives, ${\tilde{v}}_{n}^{-}$ represents the minimum value of *i* for all alternatives, whilst *B* and *C* denote the positive and negative ideal solutions, respectively.

At this stage, the separation distances between all the alternatives and the FPIS and FNIS values were determined based on the Euclidean distance (furthest and shortest) for the solutions [30]. The separation distance was measured using Equations (12)–(14) [31]:

(12) $$S_{i}^{+}=\overset{n}{\underset{j=1}{\sum }}d\left({\tilde{v}}_{ij},{\tilde{v}}_{j}^{+}\right),\;i=1,2,\ldots \ldots ,\;m,$$

(13) $$S_{i}^{-}=\overset{n}{\underset{j=1}{\sum }}d\left({\tilde{v}}_{ij},{\tilde{v}}_{j}^{-}\right),\;i=1,2,\ldots \ldots ,\;m,$$

where $S_{i}^{+}\;$ denotes the total separation distance for the FPIS value and $S_{i}^{-}\;$ denotes the total separation distance for the FNIS value for each criterion.

(14) $$d_{v}\left({\tilde{M}}_{1},{\tilde{M}}_{2}\right)=\sqrt{\frac{1}{3}\left[{\left(a_{1}-a_{2}\right)}^{2}+{\left(b_{1}-b_{2}\right)}^{2}+{\left(c_{1}-c_{2}\right)}^{2}\right]},$$

where $d_{v}\left({\tilde{M}}_{1},{\tilde{M}}_{2}\right)$ can be defined as the separation distance between the two fuzzy numbers (${\tilde{M}}_{1}\;\mathrm{and}\;{\tilde{M}}_{2}$), which was given to the two different TFN elements of ($a_{1},\;b_{1},\;c_{1}$) and ($a_{2},\;b_{2},\;c_{2}$).

Once the FPIS and FNIS separation measures had been evaluated for each criterion, the total summation of the FPIS and FNIS values for each criterion and alternative was determined. At the end, the ranking determining the best dimple core design was evaluated using Closeness Coefficient (*CC_i_*) analysis, as shown in Equation (15) [29]:

(15) $$CC_{i}=\frac{S_{i}^{-}}{\left(S_{i}^{-}+S_{i}^{+}\right)},\;0\leq R_{i}^{*}\leq 1\;$$

**Figure 8:**
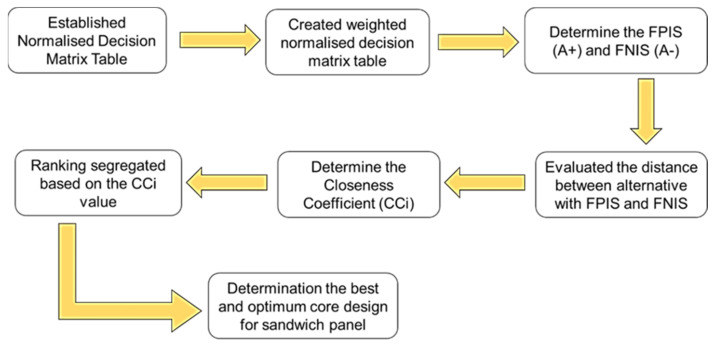
The steps to determine the best core design selection using the F-TOPSIS method.

When the final analysis of *CC_i_* had been evaluated and determined, the ranking between all the alternatives was determined. According to the existing literature, any ranking value that is near 1.0 would become the highest rank of all the alternatives and the most favourable of any alternatives [29,31]. A lower value near 0.0 would be the least favourable for selection in the analysis. Therefore, through the simulation analysis using FEA and the combination of F-MCDM, the best core design configuration alternative could be determined. Figure 8 illustrates the process flow for determining the best core design configuration using F-TOPSIS.

## 3. Results and Discussion

### 3.1. FEA under Four-Point Bending Simulations

**Figure 9:**
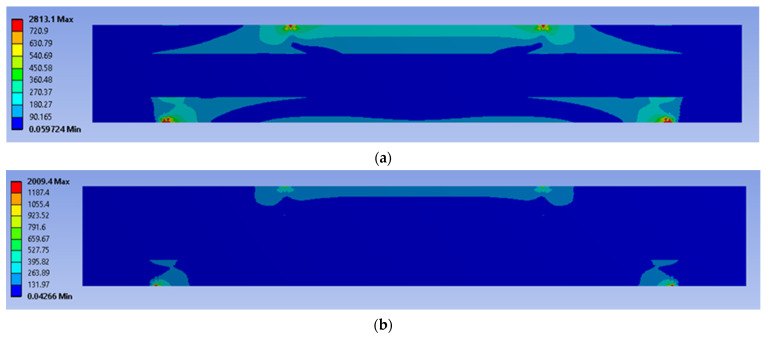
The stress distribution on a simulated panel under cyclic loading conditions (units in MPa): (**a**) 70%, (**b**) 50%.

**Figure 10:**
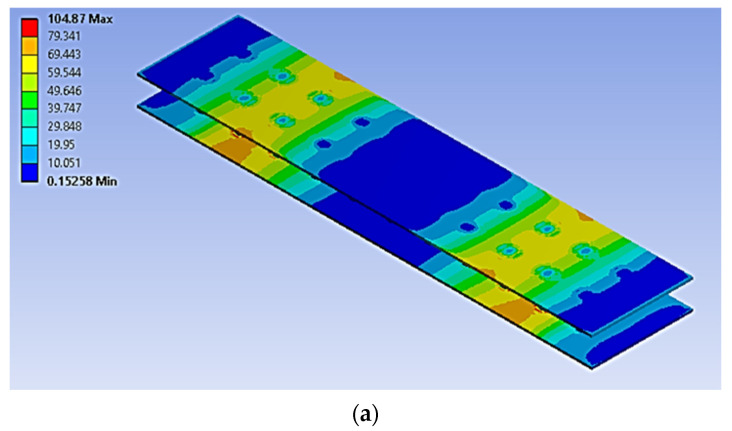

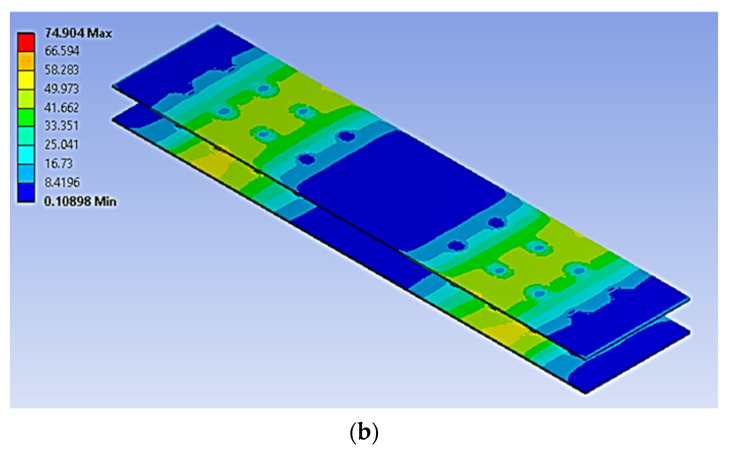
The stress distribution on the bonding area of a simulated panel under cyclic loading conditions (units in MPa): (**a**) 70%, (**b**) 50%.

**Figure 11:**
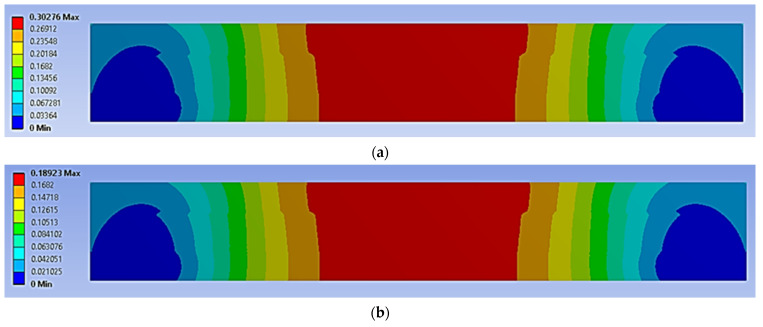
The permanent deformation experienced by a simulated panel under cyclic loading conditions (units in mm): (**a**) 70%, (**b**) 50%.

**Figure 12:**
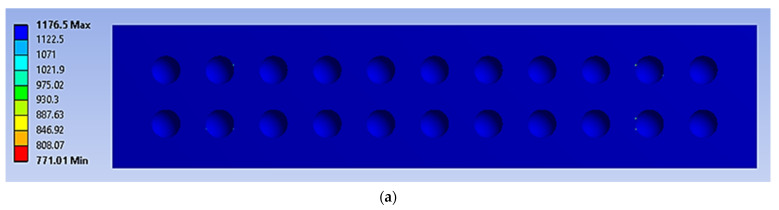

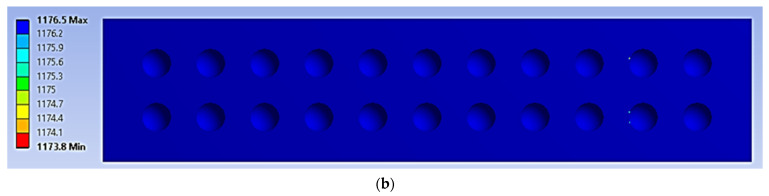
The life cycle (denoted as N_f_), at the core panel of a simulated panel under cyclic loading conditions (units in cycle): (**a**) 70%, (**b**) 50%.

**Figure 13:**
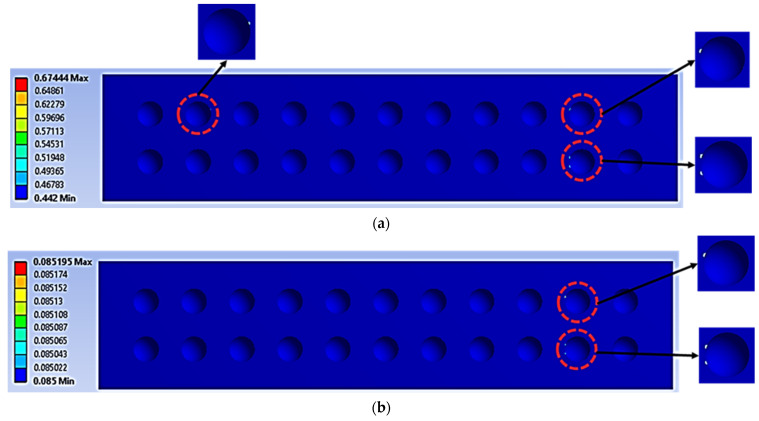
The maximum damage points region (indicated with red circles—see the zoom-in) on the core panel under cyclic loading conditions of: (**a**) 70%, (**b**) 50% (no unit for the damage value as the maximum damage value is equal to 1).

The geometrical panel used four different types of dimple core design configuration, denoted as alternative elements. Thus, this study focuses on selecting the best core design configuration, based on mechanical characteristics such as the stress distribution, permanent deformation, fatigue life and damage. These mechanical characteristics were used to justify the delamination resistance between the panel layer and justify the structural integrity of the sandwich panel [32]. Figure 9, Figure 10, Figure 11, Figure 12 and Figure 13 show that factors such as the stress distribution, permanent deformation, fatigue life and highest damage point at the dimple region played a significant role in determining the mechanical characteristics, especially at the joining area of the sandwich panel [33]. All these factors can contribute to catastrophic failures in terms of the bonding integrity and delamination phenomenon when subjected to extreme conditions, such as cyclic loading conditions [34,35]. Therefore, the FEA output was categorised into nine critical criteria for assessing the mechanical performance of a sandwich panel, as shown in Table 5 and Table 6. The output values listed in these tables indicate that the mechanical performance of all the alternative core designs varied and it was difficult to determine the best core design configuration since various factors contributed to the mechanical performance of the sandwich panel. Therefore, further analysis is required to unravel the intertwined data from the various contributory criteria and provide the best selection for the core design configuration.

### 3.2. Evaluation of Fuzzified Weightages for Critical Criteria Using F-AHP

Based on the FEA output shown in Table 5 and Table 6, the pairwise comparison matrix (PCMs) table was created according to the Saaty’s Scale. By developing the PCMs table, the relationship between the various critical criteria could be investigated and the weightages for each criterion could be determined [16,18]. To ensure the information data in the PCMs table was not biased and was acceptable in terms of inconsistency, consistency checking analysis was done [36]. From the literature, the consistency ratio value, denoted as CR, should be equal to or less than 0.1, which would mean the PCMs table was acceptable and had proved its trustworthiness [28].

Table 7 shows the developed PCMs table with nine critical criteria. At this stage, all the values in the PCMs table were evaluated and their consistency index, denoted as CI, was checked using Equation (2); this was found to be 0.1387. Further analysis done using Equation (3) determined the consistency ratio, denoted as CR. The CR value for the PCMs table was found to be 0.0957, which is less than 0.1, as shown in Table 8. The table indicates that the PCMs table had been proven free of any bias perception and was acceptable for the subsequent analysis.

Once the PCMs table had been created and justified, the next step was to convert the PCMs information data in Table 7 by inserting the fuzzy elements according to Table 4. The TFNs were infused on all the PCMs values in Table 7 using Equations (4) and (5). The fuzzified pairwise comparison matrix (F-PCMs) table could then be created, as shown in Table 9.

As Table 9 illustrates, the fuzzified weights for all the criteria were evaluated based on the geometric mean value concept. Equation (5) was used to determine the fuzzified geometric mean value for each criterion in the F-AHP analysis. The inversed evaluation of the summation vector for all the criteria in the fuzzified geometric mean values was performed using Equation (7). Hence, Equation (8) revealed that all the fuzzified weightages of all the criteria were determined at the lower, middle and upper values of the fuzzy elements, as shown in Table 10.

The full analysis in the F-AHP stage indicated that the PCMs table (shown in Table 7) had been justified and proved the consistency of the values from any point of bias and perception. The PCMs crisp values in Table 7 were then converted into the TFNs element, as shown in Table 9. The fuzzified weightages for each critical criterion were determined, as shown in Table 10. The first analysis of crisp PCM values is highly important as it ensures the robustness of the information data before it to be infused with the fuzzy number’s element [11,13]. Therefore, all the fuzzified weightages for all the criteria were used in the F-TOPSIS analysis to define the optimum core design configuration.

### 3.3. Optimum Core Design Determination Using F-TOPSIS

In Section 3.3, the best core design configuration selection was determined using the F-TOPSIS method. Using this method, the fuzzified weightages for each criterion that had been justified through the F-AHP method, as shown in Section 3.2, were used and multiplied with the normalised decision matrix table gathered from the FEA results. Hence, using Equation (9), the specific normalised decision matrix table with fuzzy elements was developed with the lower, middle and upper values, as shown in Table 11 and Table 12. As both tables illustrate, these were expected values since the Triangular Fuzzy Numbers (TFNs) element was used in both the F-AHP and F-TOPSIS methods to produce accurate and stable final results [27,30]. All these values (lower, middle and upper) were then used to analyse and obtain the FPIS and FNIS values, as shown in Table 13 and Table 14; they were determined using Equations (10)–(13).

However, it was preferable that some criterion values—such as those of the deformation and damage analysis in the dimple region under CAL and VAL loading conditions—were at their lowest value due to their significant effect on the sandwich panel. For example, high values of total deformation and core panel damage would expose the sandwich panel to several catastrophic failures, such as debonding, delamination, buckling and face yielding [37,38,39]. Therefore, the selection of positive- and negative-ideal solution values depends on the preferable performance of a sandwich panel (either most preferable or less preferable), which impacts the mechanical performance of a sandwich panel in terms of structural integrity [40,41].

**Figure 14:**
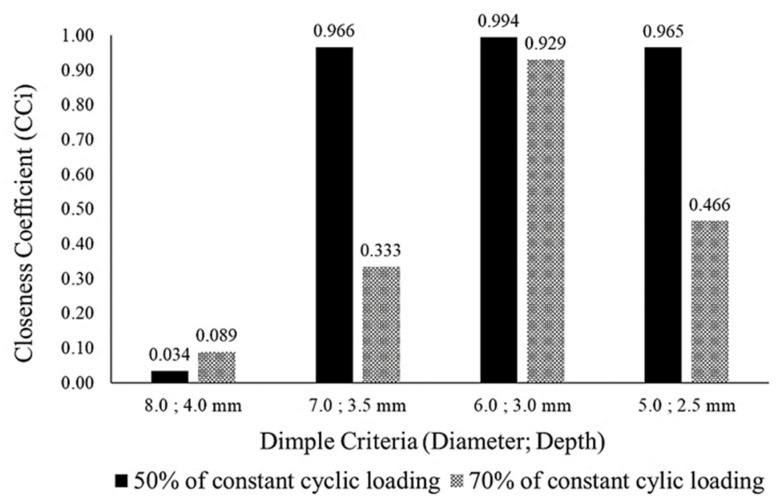
The ranking (read from highest to lowest value) between the dimple core design alternatives under cyclic loading conditions of 50% and 70%.

As Table 13 and Table 14 indicate, the summations of the FPIS and FNIS values for each alternative core design were determined using Equations (12)–(14), as shown in Table 15. The FPIS and FNIS were evaluated using the shortest and furthest distance between the ideal solutions [30]. Based on the FPIS and FNIS summation, the ranking between all the alternative hemispherical core designs was evaluated using Equation (15), and the closeness coefficient (CCi) was determined. As shown in Figure 14, the alternative core configuration with a 6.0 mm diameter and a 3.0 mm depth was the highest ranked under both 70% and 50% of cyclic loading conditions, producing values of 0.9294 and 0.9937, respectively. The alternative core configuration with an 8.0 mm diameter and a 4.0 mm depth was the lowest ranked under both 70% and 50% of cyclic loading conditions, producing values of 0.0886 and 0.0337. In the existing literature, a CCi value near 1.0 has been indicated as the most preferable alternative and the optimum selection to use for certain applications [29,31]. Therefore, it can be indicated that the core configuration with a 6.0 mm diameter and a 3.0 mm depth was the best core configuration to use as the main core under cyclic loading conditions.

**Figure 15:**
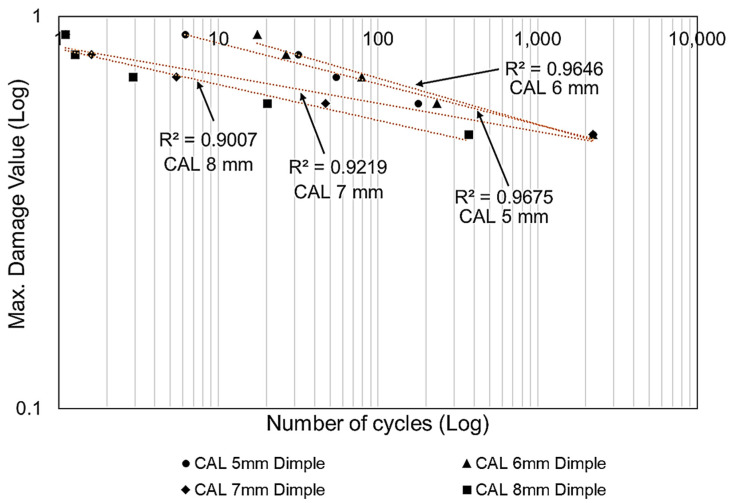
The model of damage in a logarithmic scale showing the maximum damage to the hotspot dimple region against the number of stress life cycles for various dimple core configurations under constant cyclic loading conditions.

The FEA and hybrid MCDM results were verified by modelling the damage plot in a logarithmic scale, as shown in Figure 15. In this damage plot, the highest damage distribution in the critical regions of the dimple cores were analysed and plotted against the life cycle distribution. This damage plot would be useful to determine the performance of sandwich panels based on the core configuration and loading conditions [10,23]. The dimple core configuration with a 6.0 mm diameter and a 3.0 mm depth showed the highest life cycle distribution under cyclic loading conditions. The dimples with diameters of 7.0 and 8.0 mm and depths of 3.5 and 4.0 mm showed vulnerability to early delamination since they had lower life cycle distributions than the others. This highlighted that the core design with hemispherical dimples of small sizes and dimensions tended to perform better in terms of fatigue damage and life distribution than hemispherical dimple configurations with a large size and large dimensions.

## 4. Conclusions

An investigation was conducted into different alternative core designs using various failure criteria that contribute to the performance of a sandwich panel. A simulation analysis using a combination of F-AHP and F-TOPSIS was conducted to evaluate the performance of the sandwich panel by identifying the critical criterion and determining the optimum dimple core configuration. At the F-AHP stage, the fuzzified weightages for all criteria were evaluated and the robustness of the PCMs table was checked. The results showed that the *CR* value of 0.0957 was below the cut-off value of 0.1, meaning that it was free from any inconsistency and bias perception. The main conclusions are as follows:

1. F-TOPSIS indicated that dimple core with a 6.0 mm diameter and a 3.0 mm depth was ranked the highest of the alternatives, with *CCi* values of 0.9294 and 0.9937 under conditions of 70% and 50% of cyclic loading.
2. The largest dimple core configuration with an 8.0 mm diameter and a 4.0 mm depth performed the worst, having the lowest life cycle of all the alternatives and exhibiting the highest potential to experience early delamination phenomena under cyclic loading conditions.
3. The combination of the FEA and fuzzy-hybrid MCDM results was verified using damage plot modelling in a logarithmic scale, which showed that the diameter dimple of 6.0 mm with a depth of 3.0 mm proved to be the optimum core design, with the highest life cycle distribution under constant cyclic loading conditions.
4. The damage plot modelling in Figure 15 shows that all the coefficient of determination (*R*^2^) analysis values for the sandwich panel with various dimple core configurations were above 0.90, meaning that the final results were accepted and convincing.
5. It can be concluded that a small hemispherical dimple core design has a significant effect on the sandwich panel performance and produces better delamination resistance.
6. This approach is a useful way to evaluate and justify the best core configuration alternative using various criteria that contribute to the performance of a sandwich panel to enable highly accurate and effective decisions (with a consistency sensitivity value of 0.0957 and R^2^ > 0.90).

## Figures and Tables

**Table 1:** The nine main failure criteria, segregated from the simulation results.

List	The Main Failure Criteria
C1	Maximum von Mises Stress
C2	Maximum Shear Stress
C3	Total Deformation
C4	Maximum von Mises Stress on Bonding Area
C5	Maximum Shear Stress on Bonding Area
C6	Fatigue Life at Core Panel (CAL)
C7	Highest Damage Value at Core Panel (CAL)
C8	Fatigue Life at Core Panel (VAL)
C9	Highest Damage Value at Core Panel (VAL)

**Table 2:** The total number of nodes and elements for the geometrical three-dimensional sandwich panel models with different core configurations.

	Alternative Dimple Core Designs (Diameter; Depth) in mm			
	(A1) 8.0; 4.0	(A2) 7.0; 3.5	(A3) 6.0; 3.0	(A4) 5.0; 2.5
**Total Nodes**	1,507,227	1,497,846	1,491,058	1,482,584
**Total Elements**	760,999	758,222	756,443	753,732

**Table 3:** The random index (*RI*) value by [27].

n	Random Index
2	0.00
3	0.58
4	0.90
5	1.12
6	1.24
7	1.32
8	1.41
9	1.45

**Table 4:** Saaty’s Scale for constructing the triangular F-PCMs table by [28].

Linguistic Variables	Crisp Saaty’s Scale	Fuzzified AHP Scale	
		Triangular Fuzzy Scale	Reciprocal Triangular Fuzzy Scale
Equally Important	1	(1, 1, 1)	(1, 1, 1)
Equally to Moderately Important	2	(1, 2, 3)	(1/3, 1/2, 1)
Moderately Important	3	(2, 3, 4)	(1/4, 1/3, 1/2)
Moderately to Strongly Important	4	(3, 4, 5)	(1/5, 1/4, 1/3)
Strongly Important	5	(4, 5, 6)	(1/6, 1/5, 1/4)
Strongly to Very Strongly Important	6	(5, 6, 7)	(1/7, 1/6, 1/5)

**Table 5:** FEA results for all the alternative core designs at 70% of cyclic loading conditions.

Failure Criteria from FEA	Alternative Core Designs (Diameter; Depth), mm			
	(A1) 8.0; 4.0	(A2) 7.0; 3.5	(A3) 6.0; 3.0	(A4) 5.0; 2.5
**C1**	2814.9	2808.2	2831.4	2798.9
**C2**	1483.2	1479.6	1494.5	1474.7
**C3**	0.275	0.269	0.305	0.262
**C4**	111.3	110.7	98.4	96.0
**C5**	63.9	63.4	55.7	54.1
**C6**	1,310,700	1,635,400	2,000,900	1,992,400
**C7**	0.6744	0.6860	0.0009	0.6908
**C8**	2,640,000	2,830,000	2,990,000	2,990,000
**C9**	0.7574	0.6707	0.6014	0.6688

**Table 6:** FEA results for all the alternative core designs under cyclic loading conditions of 50%.

Failure Criteria from FEA	Alternative Core Designs (Diameter; Depth), mm			
	(A1) 8.0; 4.0	(A2) 7.0; 3.5	(A3) 6.0; 3.0	(A4) 5.0; 2.5
**C1**	2026.5	2016.2	2009.4	2003.6
**C2**	1066.8	1061.7	1058.3	1055.4
**C3**	0.1958	0.1918	0.1892	0.1871
**C4**	74.9	72.8	74.9	71.9
**C5**	42.5	40.2	43.1	40.5
**C6**	1,995,460	2,000,900	2,000,900	2,000,900
**C7**	0.0852	0.0008	0.0008	0.0008
**C8**	2,991,400	2,992,900	2,993,100	2,993,100
**C9**	0.0034	0.0033	0.0030	0.0030

**Table 7:** The developed PCMs table based on the Saaty’s Scale in Table 4.

Failure Criteria	C1	C2	C3	C4	C5	C6	C7	C8	C9
**C1**	1.00	3.00	3.00	3.00	2.00	0.20	0.33	0.20	0.33
**C2**	0.33	1.00	3.00	3.00	2.00	0.20	0.33	0.20	0.33
**C3**	0.33	0.33	1.00	0.33	0.33	0.33	0.33	0.33	0.33
**C4**	0.33	0.33	3.00	1.00	2.00	0.33	0.50	0.33	0.50
**C5**	0.50	0.50	4.00	0.50	1.00	0.33	0.50	0.33	0.50
**C6**	5.00	5.00	4.00	3.00	3.00	1.00	4.00	0.50	2.00
**C7**	3.00	3.00	3.00	2.00	2.00	0.33	1.00	0.20	0.50
**C8**	5.00	5.00	4.00	3.00	3.00	2.00	5.00	1.00	3.00
**C9**	3.00	3.00	3.00	2.00	2.00	0.50	2.00	0.33	1.00

**Table 8:** The sensitivity checking procedure for all the PCMs values in Table 7.

Failure Criteria	Weighted Sum Value (λ)	Weightage of Each Criterion	Ratio of λ to Weightage for Each Criterion	Determination of *CI* and *CR*
**C1**	0.798	0.075	10.595	
**C2**	0.630	0.059	10.674	The consistency index was determined as follows:
**C3**	0.308	0.030	10.186	$CI=\frac{\lambda_{max}-n}{n-1},$
**C4**	0.582	0.057	10.259	
**C5**	0.551	0.055	10.034	*CI* = (10.110 − 9)/(9 − 1) = 0.1387
**C6**	2.141	0.219	9.763	The consistency ratio was determined as follows:
**C7**	0.991	0.099	10.051	$CR=\frac{CI}{RI},$
**C8**	2.728	0.274	9.943	*CR* = (0.1387)/1.45 = 0.096
**C9**	1.247	0.131	9.482	
		**Average λ Max**	**10.110**	***CR* < 0.1;** **Accept and Far from Bias Perception**

**Table 9:** The developed F-PCMs table based on the Saaty’s Scale in Table 4.

Failure Criteria	F-C1	F-C2	F-C3	F-C4	F-C5	F-C6	F-C7	F-C8	F-C9
**F-C1**	1, 1, 1	2, 3, 4	3, 4, 5	2, 3, 4	1, 2, 3	1/6, 1/5, 1/4	1/5, 1/4, 1/3	1/6, 1/5, 1/4	1/5, 1/4, 1/3
**F-C2**	1/4, 1/3, 1/2	1, 1, 1	2, 3, 4	2, 3, 4	1, 2, 3	1/6, 1/5, 1/4	1/5, 1/4, 1/3	1/6, 1/5, 1/4	1/5, 1/4, 1/3
**F-C3**	1/5, 1/4, 1/3	1/4, 1/3, 1/2	1, 1, 1	1/4, 1/3, 1/2	1/5, 1/4, 1/3	1/5, 1/4, 1/3	1/4, 1/3, 1/2	1/5, 1/4, 1/3	1/4, 1/3, 1/2
**F-C4**	1/4, 1/3, 1/2	1/4, 1/3, 1/2	2, 3, 4	1, 1, 1	1, 2, 3	1/4, 1/3, 1/2	1/3, 1/2, 1	1/4, 1/3, 1/2	1/3, 1/2, 1
**F-C5**	1/3, 1/2, 1	1/3, 1/2, 1	3, 4, 5	1/3, 1/2, 1	1, 1, 1	1/4, 1/3, 1/2	1/3, 1/2, 1	1/4, 1/3, 1/2	1/3, 1/2, 1
**F-C6**	4, 5, 6	4, 5, 6	3, 4, 5	2, 3, 4	2, 3, 4	1, 1, 1	3, 4, 5	1/3, 1/2, 1	1, 2, 3
**F-C7**	3, 4, 5	3, 4, 5	2, 3, 4	1, 2, 3	1, 2, 3	1/5, 1/4, 1/3	1, 1, 1	1/6, 1/5, 1/4	1/3, 1/2, 1
**F-C8**	4, 5, 6	4, 5, 6	3, 4, 5	2, 3, 4	2, 3, 4	1, 2, 3	4, 5, 6	1, 1, 1	2, 3, 4
**F-C9**	3, 4, 5	3, 4, 5	2, 3, 4	1, 2, 3	1, 2, 3	1/3, 1/2, 1	1, 2, 3	1/4, 1/3, 1/2	1, 1, 1

**Table 10:** The fuzzified weights for each criterion for lower, middle and upper values.

Failure Criteria	Fuzzified Geometric Mean Value			Fuzzified Weights		
	Lower	Middle	Upper	Lower	Middle	Upper
**F-C1**	0.6190	0.8265	1.0584	0.0404	0.0724	0.1291
**F-C2**	0.4696	0.6271	0.8195	0.0306	0.0549	0.1000
**F-C3**	0.2641	0.3314	0.4510	0.0172	0.0290	0.0550
**F-C4**	0.4569	0.6420	0.9685	0.0298	0.0562	0.1182
**F-C5**	0.4510	0.6218	1.0251	0.0294	0.0545	0.1251
**F-C6**	1.7935	2.4840	3.2738	0.1169	0.2176	0.3994
**F-C7**	0.8363	1.1904	1.6156	0.0545	0.1043	0.1971
**F-C8**	2.2597	3.1072	3.8972	0.1473	0.2721	0.4755
**F-C9**	1.0461	1.5874	2.2275	0.0682	0.1390	0.2718

**Table 11:** The specific normalised decision matrix table with fuzzy elements for all the main alternatives under 70% of cyclic loading conditions.

Failure Criteria	Main Alternatives											
	A1			A2			A3			A4		
	* L	* M	* U	* L	* M	* U	* L	* M	* U	* L	* M	* U
**F-C1**	0.0202	0.0362	0.0646	0.0201	0.0361	0.0644	0.0203	0.0364	0.0650	0.0201	0.0360	0.0642
**F-C2**	0.0153	0.0275	0.0500	0.0153	0.0274	0.0499	0.0154	0.0277	0.0504	0.0152	0.0273	0.0497
**F-C3**	0.0085	0.0143	0.0272	0.0083	0.0140	0.0266	0.0094	0.0159	0.0302	0.0081	0.0137	0.0259
**F-C4**	0.0159	0.0300	0.0630	0.0158	0.0298	0.0627	0.0140	0.0265	0.0557	0.0137	0.0259	0.0544
**F-C5**	0.0158	0.0293	0.0672	0.0157	0.0290	0.0667	0.0138	0.0255	0.0586	0.0134	0.0248	0.0569
**F-C6**	0.0436	0.0811	0.1489	0.0544	0.1012	0.1858	0.0665	0.1238	0.2273	0.0663	0.1233	0.2263
**F-C7**	0.0311	0.0594	0.1123	0.0316	0.0604	0.1142	0.0000	0.0001	0.0001	0.0318	0.0608	0.1150
**F-C8**	0.0679	0.1253	0.2190	0.0727	0.1344	0.2347	0.0769	0.1420	0.2480	0.0769	0.1420	0.2480
**F-C9**	0.0382	0.0778	0.1521	0.0338	0.0689	0.1347	0.0303	0.0618	0.1207	0.0337	0.0687	0.1343

* L = Lower, M = Middle, U = Upper.

**Table 12:** The specific normalised decision matrix table with fuzzy elements for all the main alternatives at 50% of cyclic loading conditions.

Failure Criteria	Main Alternatives											
	A1			A2			A3			A4		
	* L	* M	* U	* L	* M	* U	* L	* M	* U	* L	* M	* U
**F-C1**	0.0203	0.0364	0.0650	0.0202	0.0362	0.0646	0.0201	0.0361	0.0644	0.0201	0.0360	0.0642
**F-C2**	0.0154	0.0276	0.0503	0.0153	0.0275	0.0500	0.0153	0.0274	0.0499	0.0152	0.0273	0.0497
**F-C3**	0.0088	0.0149	0.0282	0.0086	0.0146	0.0276	0.0085	0.0144	0.0273	0.0084	0.0142	0.0269
**F-C4**	0.0151	0.0286	0.0601	0.0147	0.0278	0.0584	0.0151	0.0286	0.0601	0.0146	0.0275	0.0578
**F-C5**	0.0150	0.0278	0.0639	0.0142	0.0263	0.0604	0.0152	0.0282	0.0647	0.0143	0.0265	0.0609
**F-C6**	0.0584	0.1086	0.1993	0.0585	0.1089	0.1999	0.0585	0.1089	0.1999	0.0585	0.1089	0.1999
**F-C7**	0.0545	0.1042	0.1971	0.0005	0.0010	0.0020	0.0005	0.0010	0.0020	0.0005	0.0010	0.0020
**F-C8**	0.0736	0.1360	0.2376	0.0737	0.1361	0.2378	0.0737	0.1361	0.2378	0.0737	0.1361	0.2378
**F-C9**	0.0341	0.0695	0.1359	0.0341	0.0695	0.1359	0.0341	0.0695	0.1359	0.0341	0.0695	0.1359

* L = Lower, M = Middle, U = Upper.

**Table 13:** FPIS and FNIS values at 70% of cyclic loading conditions.

Failure Criteria	Si^+^			Si^−^		
	* L	* M	* U	* L	* M	* U
**F-C1**	0.0203	0.0364	0.0650	0.0201	0.0360	0.0642
**F-C2**	0.0154	0.0277	0.0504	0.0152	0.0273	0.0497
**F-C3**	0.0081	0.0137	0.0259	0.0094	0.0159	0.0302
**F-C4**	0.0159	0.0300	0.0630	0.0137	0.0259	0.0544
**F-C5**	0.0158	0.0293	0.0672	0.0134	0.0248	0.0569
**F-C6**	0.0665	0.1238	0.2273	0.0436	0.0811	0.1489
**F-C7**	0.0000	0.0001	0.0001	0.0318	0.0608	0.1150
**F-C8**	0.0769	0.1420	0.2480	0.0679	0.1253	0.2190
**F-C9**	0.0303	0.0618	0.1207	0.0382	0.0778	0.1521

* L = Lower, M = Middle, U = Upper.

**Table 14:** FPIS and FNIS values at 50% of cyclic loading conditions.

Failure Criteria	Si^+^			Si^−^		
	* L	* M	* U	* L	* M	* U
**F-C1**	0.0203	0.0364	0.0650	0.0201	0.0360	0.0642
**F-C2**	0.0154	0.0276	0.0503	0.0152	0.0273	0.0497
**F-C3**	0.0084	0.0142	0.0269	0.0088	0.0149	0.0282
**F-C4**	0.0151	0.0286	0.0601	0.0146	0.0275	0.0578
**F-C5**	0.0152	0.0282	0.0647	0.0142	0.0263	0.0604
**F-C6**	0.0585	0.1089	0.1999	0.0584	0.1086	0.1993
**F-C7**	0.0005	0.0010	0.0020	0.0545	0.1042	0.1971
**F-C8**	0.0737	0.1361	0.2378	0.0736	0.1360	0.2376
**F-C9**	0.0341	0.0695	0.1359	0.0341	0.0695	0.1359

* L = Lower, M = Middle, U = Upper.

**Table 15:** Summation of FPIS and FNIS through all the main alternatives to determine the dimple core configuration ranking, based on the Closeness Coefficient analysis.

Loading Conditions	Alternatives (Diameter; Depth), mm	Total Si^+^	Total Si^−^	Closeness Coefficient (CCi)	Ranking
Under 70% of cyclic loading conditions	(A1) 8.0; 4.0	0.1708	0.0166	0.0886	4
	(A2) 7.0; 3.5	0.1250	0.0624	0.3331	3
	**(A3) 6.0; 3.0**	0.0132	0.1742	0.9294	**1**
	(A4) 5.0; 2.5	0.1001	0.0873	0.4657	2
Under 50% of cyclic loading conditions	(A1) 8.0; 4.0	0.1331	0.0046	0.0337	4
	(A2) 7.0; 3.5	0.0047	0.1330	0.9656	2
	**(A3) 6.0; 3.0**	0.0009	0.1368	0.9937	**1**
	(A4) 5.0; 2.5	0.0048	0.1329	0.9650	3

## Data Availability

The processed data/material required to reproduce these findings cannot be shared at this time as the data also forms part of an ongoing study.
